# Urinary CD133^+^ Extracellular Vesicles Are Decreased in Kidney Transplanted Patients with Slow Graft Function and Vascular Damage

**DOI:** 10.1371/journal.pone.0104490

**Published:** 2014-08-06

**Authors:** Veronica Dimuccio, Andrea Ranghino, Loredana Praticò Barbato, Fabrizio Fop, Luigi Biancone, Giovanni Camussi, Benedetta Bussolati

**Affiliations:** 1 Department of Molecular Biotechnology and Health Sciences, University of Torino, Torino, Italy; 2 Department of Medical Sciences, University of Torino, Torino, Italy; 3 Immunogenetics and Transplant Biology Service, Città della Salute e della Scienza Hospital, Torino, Italy; 4 Division of Nephrology, Dialysis and Transplantation, Città della Salute e della Scienza Hospital, Torino, Italy; Universtiy of Maryland Schoool of Medicine, United States of America

## Abstract

Extracellular vesicles (EVs) present in the urine are mainly released from cells of the nephron and can therefore provide information on kidney function. We here evaluated the presence of vesicles expressing the progenitor marker CD133 in the urine of normal subjects and of patients undergoing renal transplant. We found that EV expressing CD133 were present in the urine of normal subjects, but not of patients with end stage renal disease. The first day after transplant, urinary CD133^+^ EVs were present at low levels, to increase thereafter (at day 7). Urinary CD133^+^ EVs significantly increased in patients with slow graft function in respect to those with early graft function. In patients with a severe pre-transplant vascular damage of the graft, CD133^+^ EVs did not increase at day 7. At variance, the levels of EVs expressing the renal exosomal marker CD24 did not vary in the urine of patients with end stage renal disease or in transplanted patients in respect to controls. Sorted CD133^+^ EVs were found to express glomerular and proximal tubular markers. These data indicate that urinary CD133^+^ EVs are continuously released during the homeostatic turnover of the nephron and may provide information on its function or regenerative potential.

## Introduction

Extracellular vesicles (EVs) are circular fragments of membrane released from the endosomal compartment or shed from the surface membranes of most cell types [1]. EVs express surface receptors and contain proteins and extracellular RNA shared in great part with the cell of origin and their presence has been identified in all biological fluids [1], [2]. In the urine, EVs are considered to mainly derive from cells of the nephron, with a minimal contribution of the lower urinary collecting system cells [3]. Indeed, proteomic analysis of urinary EVs identified membrane and cytosolic proteins, which have characteristics of renal tubule epithelial cells of different nephron segments and of podocytes [3], [4]. Urinary EVs can therefore provide quantitative and qualitative information on the state of the kidney [5], thus suggesting their use as biomarkers in renal pathology.

After kidney transplantation from deceased donor, ischemia-reperfusion injury of the graft causes renal tubular epithelial cell necrosis and apoptosis leading to loss of organ function [6], [7]. A slow or delayed graft function may affect approximately 25% of transplant recipients, causing a significant increase in early transplant-related morbidity and a long term decrease of graft survival [6]. The current approach to clinical management of episodes of renal dysfunction during the first six months after transplantation is mainly based on the results of a percutaneous allograft biopsy [7]. In addition, advances in non-invasive immune monitoring for rejection, such as measurements of signs of cellular immune response, are currently available [8]. However, non invasive tools for evaluation of renal damage and regeneration would be required to guide effective therapeutic interventions.

Intrinsic mechanisms of tissue repair and regeneration occur in the mammalian kidney to re-acquire functionality after ischemic, toxic or inflammatory insults [9]. Recent evidence indicates that cells with characteristics of progenitors, expressing the stem cell marker CD133 and lacking differentiation markers, are present in different segments of the human nephron, being localized in the Bowman capsule of glomeruli, in proximal tubules as well as in inner medullary papilla region including Henle’s loop and S3 limb segment [10]–[12]. In addition, CD133^+^ cells in the cortex increase in number after acute renal damage, suggesting their role in renal repair after injury [13]. In particular, the number of CD133^+^ cells was reported to increase in tubules of transplanted patients undergoing delayed graft function as a result of acute renal injury [13], as well as of patients with proteinuric glomerular diseases [14], [15].

Somatic stem and precursor cells of nervous and hematopoietic origin have been shown to release CD133-carrying EVs [16], [17] and their level in the spinocerebral fluids associated to pathological conditions [18], [19]. We therefore reasoned that CD133^+^ cells along the nephron could similarly release CD133-carrying EVs and that the presence of these EVs in the urine may provide information on the physiopathological state of the kidney or on its regenerative ability. Indeed, CD133-carrying EVs were previously reported in the urine of normal subjects [20]. In the present study, we aimed to better characterize the urinary CD133^+^ EVs and to assess their levels in normal subjects and in patients with chronic and acute renal dysfunction. In particular, CD133^+^ EVs were evaluated in patients with end stage renal disease and in kidney transplanted patients with early or slow graft function, at different days after transplantation.

## Materials and Methods

### Ethical statement

i) Urine samples: the study was conducted using urine samples from normal human volunteers and transplanted patients. Approval from the San Giovanni Battista Hospital Review Board was obtained, and written informed consent was given by each participant. ii) HLA typing sera from multiparous women: HLA typing sera were obtained from multiparous women by the HLA typing laboratory of our hospital following approval of the San Giovanni Battista Hospital Review Board and stored in an anonymous fashion. All participants gave written consent for their samples to be used in research. iii) kidneys from deceased donors: the evaluation of vascular lesions was performed on protocol biopsies of kidneys from deceased donors prior to transplantation. All organ donor next of kin gave written consent for use of the donor samples in research (as part of the overall protocol and consent for organ donation), as approved by the Regional Transplant Institutional Board.

### Patients

Urine samples were obtained from normal human volunteers and transplanted patients. Levels of CD133^+^ EVs were compared in transplanted patients (n = 25), in age-matched controls (n = 20) and in end stage renal disease (ESRD) patients with residual diuresis (n = 5). All patient information is presented in Table 1. In addition, in selected experiments, urinary EVs were obtained from urine pooled from normal subjects (n = 10, age: 28±3, M/F: 4/6).

**Table 1:** Characteristics of the transplanted patients, ESRD patients and normal subjects in the study.

	Early graf function (n = 13)	Slow graft function (n = 12)	
**RECIPIENTS**			
**Age, years**	53.8±14.1	56.1±12.1	ns
**Sex, (M/F)**	6/7	11/2	
**Cause of chronic** **kidney disease**	Unknown, 3; Chronicpyelonephritis, 2; APKD, 2;Gn, 5; nephroangiosclerosis, 1.	Unknown, 1; Chronic pyelonephritis,3; Gn, 5; nephroangiosclerosis, 1;diabetic nephropathy, 2;hemolytic-uremic syndrome, 1.	
**Prior treatment**			
**Hemodialysis, No (%)**	11 (84)	10 (77)	ns
**Peritoneal dialysis,** **No (%)**	2 (15.3)	3 (23)	ns
**Time on dialysis** **(months)**	51.1±48.9	71.5±126	ns
**Retransplant, No (%)**	3 (23)	3 (23)	ns
**Panel reactive** **Antibody >20%, No (%)**	2 (15.3)	1(7.7)	ns
**≥4 HLA mismatches,** **No (%)**	12 (100%)	11 (84%)	ns
**LD/DD**	0/13	1/12	ns
**Cold ischemia time, h**	14.5±5	14.1±5.5	ns
**Induction ID, No. (%)**			
**Basiliximab**	13 (100)	11 (84)	ns
**Rituximab**	0	1 (7)	ns
**No induction**	0	1 (7)	ns
**Maintenance ID,** **No. (%)**			
**Tacrolimus**	13 (100)	12 (92)	ns
**Mycophenolate mofetil**	11 (84)	12 (92)	ns
**Steroid**	13 (100)	13 (100)	ns
**Urine 24-h volume (ml)**			
**Before transplantation**	577±701	700±730	ns
**day 1 post-transplant**	2875±2799	1110±989	p = 0.02
**day 7 post-transplant**	3706±1098	3881±2025	ns
**GFR**			ns
**day 1 post-transplant**	14.2±6.5	12.8±4.3	ns
**day 7 post-transplant**	45.2±23	14.3±5.4	p = 0.0001
**DONORS**			
**Age, years**	60.9±20.5	67.3±11	ns
**ECD, No (%)**	11 (84%)	11 (84%)	ns
**GFR (ml/min)**	98.8±49	84.3±17	ns
**Hypertension, No (%)**	8 (61.5)	9 (69.2)	ns
**Diabetes, No (%)**	3 (23)	1 (7.7)	
**Histological score**			
**Glomerular sclerosis**	1.2±0.4	0.9±0.3	p = 0.04
**Tubular atrophy**	0.2±0.4	0.1±0.3	ns
**Interstitial fibrosis**	0.5±0.5	0.3±0.5	ns
**Arterial and arteriolar** **narrowing**	1.2±0.4	0.9±0.5	ns
**Final score**	3±1	2.2±0.9	p = 0.04
**ESRD patients (n = 5)**			
**Age, yrs**	54.4±17,6		
**Sex, (M/F)**	1/4		
**Cause of chronic kidney** **disease**	APKD, 2; Unknown, 1;nephroangiosclerosis, 1;Cisplatin-inducednephropathy, 1		
**Urine 24-hours volume** **(ml)**	1780±705		
**CONTROL SUBJECTS** **(n = 20)**			
**Age, yrs**	51.8±17.1		
**Sex, (M/F)**	11/9		

**ECD:** expanded criteria donors according to Crystal City Meeting criteria [35]. **GFR:** glomerular filtration rate. **Histological score:** performed on pretransplant biopsies according to a semiquantitative method [25]. **APKD:** adult polycystic kidney disease; **Gn:** glomerulonephritis; **LD:** living donor; **DD:** deceased donor. **ID:** immunosuppressive therapy. **ESRD:** end-stage kidney disease. Continuous variables are express as mean ± SD.

### Evs isolation and count

Urinary EVs from transplanted patients were collected from 80 ml of urine drained by a vescical catheter, from a 6-hours collection, at the first (D1) and the seventh (D7) day after the transplant. In selected patients (n = 6), urine was also collected after 30 days. Urine from controls (control normal subjects) and ESRD patients was obtained from 80 ml of the second morning urine or of a 6-hours collection. In addition urine was collected from normal subjects after an oral water load. After voiding, a water load of 20 ml/kg was given orally for 30 minutes. Urine was collected for 6 hours [21].

Urine was centrifuged at 2500 rpm for 15 minutes to pellet cells and Tamm-Horsfall protein. The supernatant was supplemented with protease inhibitor (Sigma) and NaN_3_ 10 mM (Fluka), filtered through a 0.8 µM filter (Millipore) and then ultracentrifuged at 100.000 g for 1 hour at 4°C. The pellet was resuspended in RPMI (Sigma) and 1% DMSO (Sigma) was added to allow freezing storage in −80°C until use.

#### EV quantification

For cytofluorimetric and Western Blot analysis, samples were quantified through a proteic Bradford assay. Urinary EVs were also analysed by nanoparticle tracking analysis (NTA), using the NanoSight LM10 system (NanoSight Ltd, Amesbury, UK), configured with a 405 nm laser and a high sensitivity digital camera system (OrcaFlash2.8, Hamamatsu C11440, NanoSight Ltd) as previously described [22]. Briefly, EVs coming from 80 ml of urine were resuspended in 100 µl of vesicle-free physiologic solution (Fresenius Kabi, Runcorn, UK). Each sample was then diluted 1∶1000 in the same solution. For this analysis, a monochromatic laser beam at 405 nm was applied to the diluted suspension of EVs. For each sample, three videos of 30 seconds duration were recorded and number of particles/ml and mean size was averaged. NTA post-acquisition settings were optimized and kept constant between samples, and each video was then analysed to give the mean, mode, and median vesicle size together with an estimation of the concentration.

### Sorting of CD133^+^ fraction

Sorting of CD133^+^ EVs was performed on urine pools obtained from the second morning urine of normal subjects. CD133^+^ EVs were isolated by magnetic cell sorting, using the CD133 MicroBead Kit applied to the MACS system (Miltenyi Biotec, Auburn, CA). Briefly, urinary EVs were collected from a total volume of 200 ml of urine. The relative pellet, obtained after the 100.000 g ultracentrifugation, was resuspended in 100 µl of ultracentrifuged vesicle-free MACS Buffer, then 100 µl of FcR Blocking Reagent and 100 µl of CD133 MicroBeads were added. This mix was incubated for 30 minutes at 4°C. Hereafter 250 µl of vesicle-free MACS buffer was added to stop the coupling reaction of the antibody to the EVs. The final sample volume of 550 µl was totally applied onto the LS-column for the EVs separation as recommended by the manufacturer’s instructions. CD133^−^ EVs, finally present in a buffer suspension of 9 ml, and the CD133^+^ EVs, present in a buffer suspension of 5 ml, were then collected through a further 100.000 g centrifuge. Both CD133^+^ and CD133^−^ EVs pellets were analyzed using Western blot analysis. For nanoparticle tracking analysis, CD133^+^ EVs were sorted using an indirect labelling that allows removal magnetic beads with the APC multisorting kit (Miltenyi). Briefly, pelleted EVs were stained with the APC conjugated CD133 mAb (1∶20 dilution, 10 minutes at 4°C), ultracentrifuged, and then sorted with the APC-multisorting kit following the manufacture’s instructions.

### Cytofluorimetric analysis

Flow cytometry analysis was performed with a FACSCalibur machine using CellQuest software (Becton Dickinson Bioscience Pharmingen, Franklyn Lake, NJ, USA). As EVs are too small for FACScan analysis, we bound EVs to surfactant-free white aldehyde/sulfate latex beads 4% w/v, 4 µm diameter (Molecular Probes, Invitrogen) [23]. We incubated 5 µl beads with 15 µg EVs for 10–15 minutes at RT and then overnight at 4°C in a final volume of 1 ml PBS-BSA 0.5%. Then the adsorbed EVs were divided in different vials and incubated with antibodies diluted 1∶50, for 15 minutes at 4°C. The adsorbed EVs were then washed and analyzed with a FACSCalibur and CellQuest software. Flow cytometry was performed using anti-human monoclonal antibodies (MAbs): APC-conjugated CD133 (AC133; Miltenyi, Auburn, CA, USA); FITC conjugated CD24, PE-conjugated CD9, PE-conjugated CD81 (Becton Dickinson) and anti-Rab5 (Santa Cruz Biotechnology, Santa Cruz, CA, USA). Comparison of urinary EVs among groups was performed by evaluating the relative amount of EVs positive for a selected marker in respect to all EVs, in conditions where the same quantity of EVs was tested (15 µg protein, corresponding roughly to 1×10^12^ urinary EVs/µg protein, as evaluated by Bradford protein analysis and NTA, respectively). This protein amount was selected by preliminary experiments using different amounts of EVs (from 1 to 50 µg,) that showed no increase in CD133 level at concentration higher than 15 µg (Figure S1). To assess vesicle binding to latex beads, CD133^+^ or CD133^−^ EVs were labelled with Vibrant Dil cell-labeling solution (Molecular Probes by Life Techonologies) and processed for fluorescence analysis. No difference was observed in CD133^+^ or CD133^−^ EV fluorescence (Figure S1).

### Western Blot

For protein analysis, both the CD133^+^ and the CD133^−^ fractions of EVs were lysed at 4°C for 30 minutes in RIPA buffer (20 nM Tris-HCl, 150 nM NaCl, 1% deoxycholate, 0.1% SDS, 1% Triton X-100, pH 7.8) supplemented with protease and phosphatase inhibitors cocktail and PMSF (Sigma). When the protein concentration was lower than 2 mg/ml a protocol was used to precipitate, and then concentrate, 10 µg of proteins [24]. Briefly, the volume corresponding to 10 µg was brought to 250 µl with deionized water, and then 800 µl of Methanol, 200 µl of Chloroform and dH2O were added. The mix was strongly vortexed and centrifuged at 12000 rpm for 2 minutes. The upper phase was removed and 600 µl of MetOH was added. After a centrifuge the upper part was removed and the pellet was left to dry under the hood. The remaining dried pellet was resuspended in a proper volume of Laemmli Buffer and the sample was loaded onto the gel. Aliquots of the cell lysates containing 10 µg proteins, as determined by the Bradford method, were run on 10% acrylamide gel SDS-PAGE under reducing conditions. The transfer of proteins onto a PVDF membrane was performed in a 7 minutes transfer program of the iBlot™ Dry Blotting System (Life Technology). The following primary antibodies were used: CD133/1 (clones AC133 and W6B3C1) (Miltenyi), rabbit polyclonal CD133 (Abcam) both at 1∶500 dilution; CD81, CD9 and CD63 (all from Becton Dickinson) at 1∶200 dilution; Megalin, Aquaporin-1 (AQ1) and Aquaporin-2 (AQ2) at 1∶100 dilution; Aminopeptidase A and CD2 Associated protein (CD2AP) (all from Santa Cruz Biotechnology) at 1∶200 dilution.

#### HLA typing sera

Sera from multiparous women were obtained by the HLA typing laboratory of our hospital. Sera characterized to contain antibodies directed against the HLA class I antigens of the donors were used in order to distinguish the origin of the extracellular vesicles. Typing sera were used at 1∶50 dilution in Western Blot experiments. The use of an irrelevant serum gave no signal (data not shown).

#### Histological evaluation of vascular lesions

Briefly, kidneys from deceased donors underwent a protocol biopsy prior to transplantation in order to quantify the severity of tissue damage according to the histological score proposed by Remuzzi et al [25]. All specimens were evaluated by the same renal pathologist. Specifically, vascular lesions defined as arterial and arteriolar narrowing were scored as follow: 0 absent (n = 7), 1 increased wall thickness but to a degree that is less than the diameter of the lumen (n = 15), 2 wall thickness that is equal or slightly greater to the diameter of the lumen (n = 3), 3 wall thickness that far exceeds the diameter of the lumen with extreme luminal narrowing or occlusion (n = 0).

### Statistical methods

Statistical analysis was performed with SPSS (IBM SPSS Statistics, vers. 12.0.0). Continuous variables are presented as median (min–max), according to their distribution. The difference between groups of these variables was analyzed with Mann-Whitney, Wilcoxon or Kruskal-Wallis test.

Categorical variables are presented as fraction and Pearson’s or for small samples Fisher’s exact test was employed to compare groups. The odds ratios (OR) were used as a measure of relative risk. Significance level for all tests was set at p<0.05.

## Results

### Detection and characterization of CD133^+^ urinary EV in normal subjects

**Figure 1:**
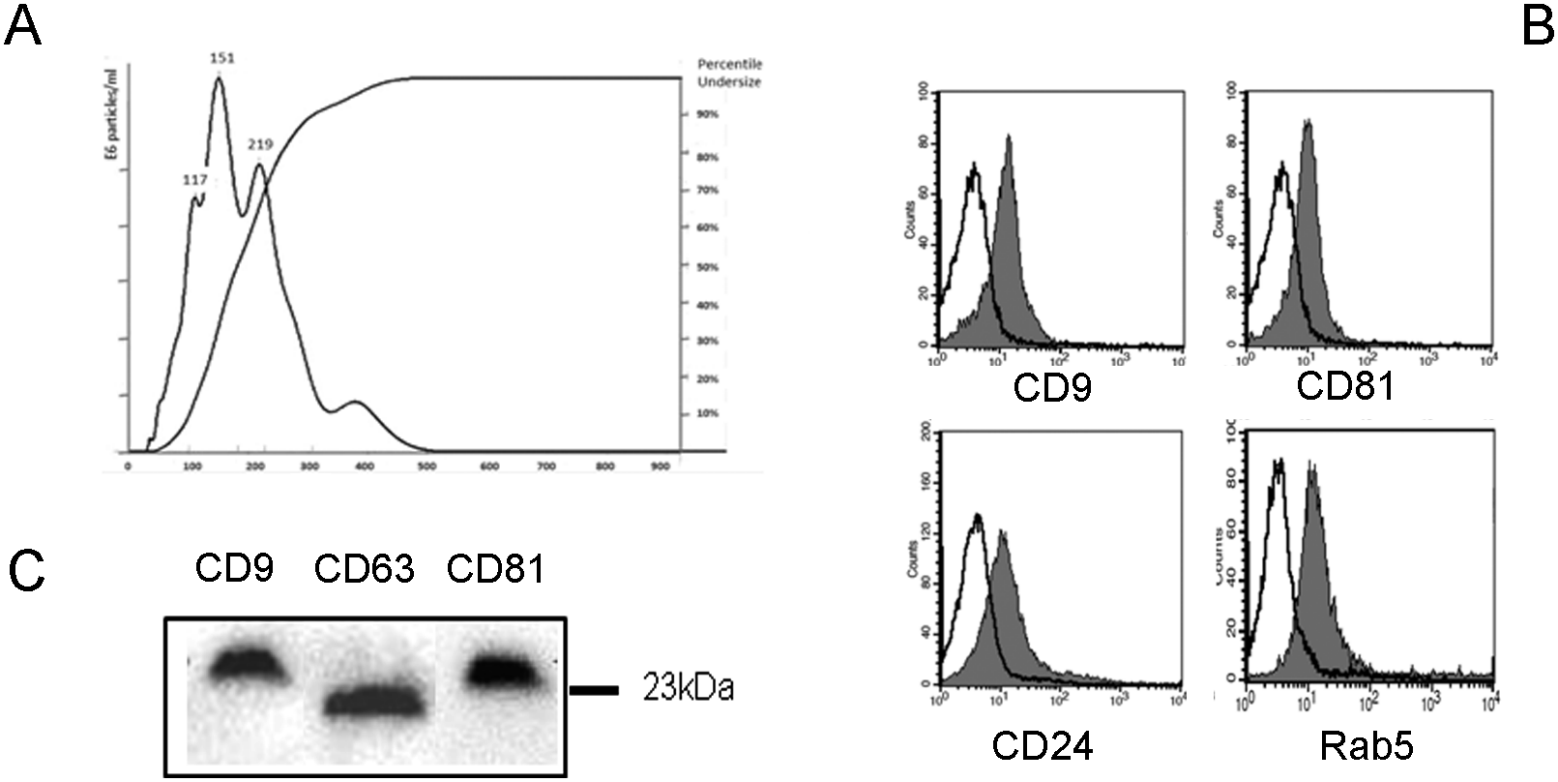
Characterization of normal urinary EVs. Panel A. NanoSight analysis of samples prepared from the urine of normal subjects showing their size distribution profile. A peak at 151**Panel B and C**. Representative images of the expression of exosome markers by urinary EVs by cytofluorimetric (B) and Western Blot analysis (C). Cytofluorimetric histograms show marker detection (grey area) and isotypic control (dark line). Five different preparations were tested with similar results.

EVs were isolated from urine obtained from normal subjects using a two-step differential centrifugation protocol based on a first low-speed centrifugation, followed by filtration trough a 0.8 µm filter to remove cells, cellular debris and apoptotic bodies. The supernatant was subsequently ultracentrifuged at 100,000 g for 1 h to sediment urinary vesicles. Urinary EVs showed a heterogenic size, as evaluated by NanoSight analysis, with a mean peak of 151 nm (Fig. 1A).

**Figure 2:**
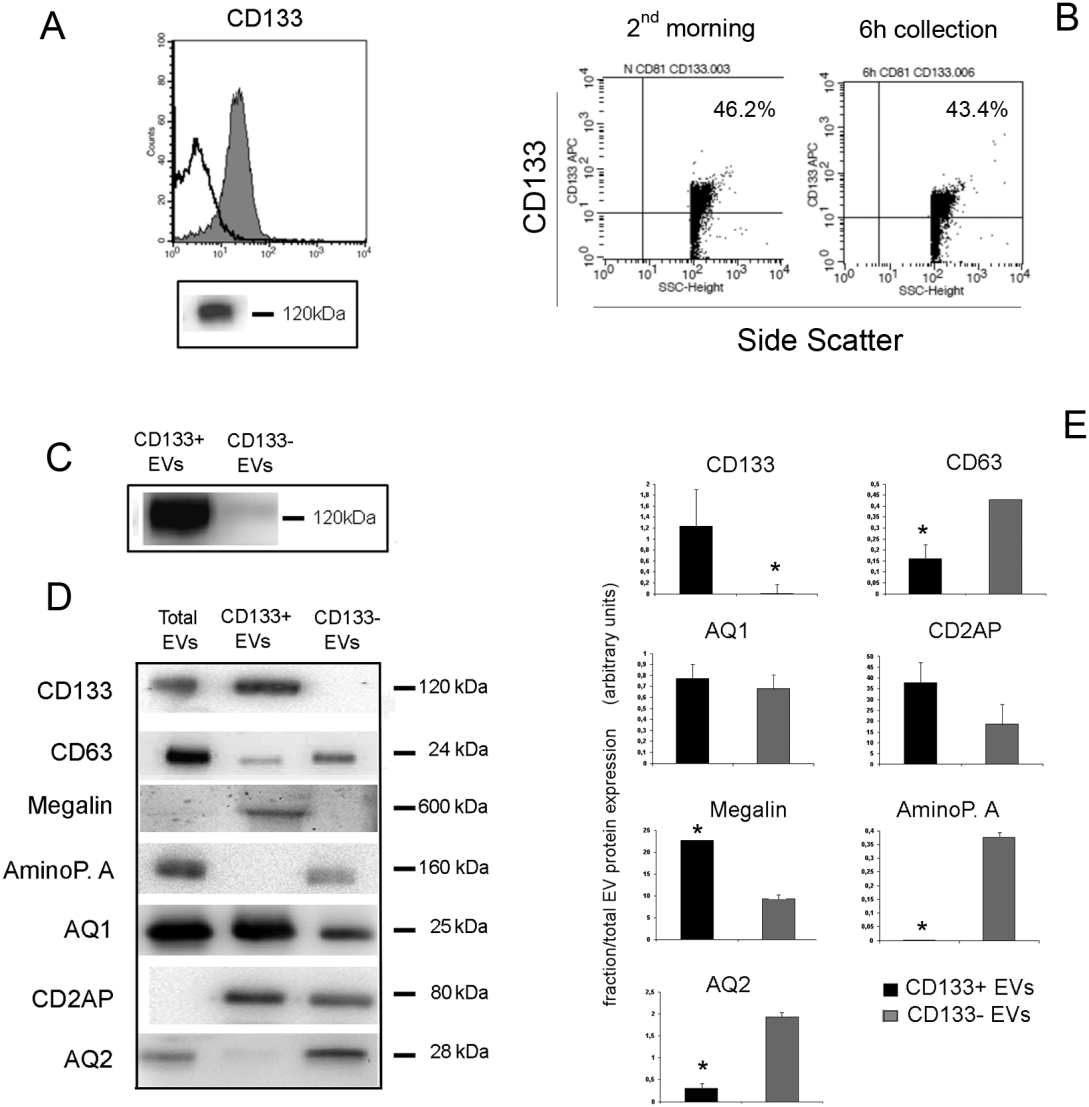
Expression of CD133 by urinary vesicles. Panel A. Representative cytofluorimetric and Western Blot (clone AC133) images of the expression of CD133 by urinary vesicles of normal subjects. The cytofluorimetric histogram shows CD133 detection (grey area) and isotypic control (dark line). **Panel B**. Scatter and fluorescence dot plots showing similar CD133^+^ EV levels in normal urine of a second morning minction or of a 6-hours collection. Plots are representative of three experiments with similar results. **Panel C**. Representative image of CD133 detection in CD133^+^ and CD133^−^ sorted EVs (clone W6B3C1). **Panel D and E**. Representative images and quantification of the expression of different nephron markers by the total urinary EVs and by CD133^+^ and CD133^−^ sorted EVs. Data are mean ± SD of at least three different experiments and report the marker expression in the sorted fractions normalized on the expression in the Total EVs. AminoP.: Aminopeptidase A, AQ: Aquaporin, CD2AP: CD2 Associated protein. * = p<0.05.

Cytofluorimetric analysis of the EVs using latex beads showed presence of typical exosome markers such as CD9, CD81 and Rab5, and of the renal vesicle marker CD24 [26] (Fig. 1B). Figure 1C shows Western Blot analysis of EVs expressing the tetraspanins CD9, CD63 and CD81. We focused our attention on CD133, a stem cell marker expressed in the kidney by progenitors cells [10]–[12] and previously described in the urinary EVs [20]. We found that CD133^+^ EVs were detectable at high levels in the urine of normal subjects using cytofluorimetric assay (Fig. 2A). No difference was observed in the levels of CD133^+^ EV according to the urine collection protocol, as the same results were obtained in the second-morning urine or in a 6 h collection urine of the same subject (Fig. 2B). By Western Blot analysis, CD133 was detected using the stem/progenitor specific antibody against the AC133 isoform, and its molecular weight was compatible with the glycosylated isoform (120 kDa) confirming that EVs expressed the stem/progenitor specific isoform of CD133 [17], [20] (Fig. 2A).

To further characterize the possible origin of CD133^+^ EVs from different nephron segments, we sorted CD133^+^ EVs and compared the expression of typical nephron markers in the CD133 positive and negative fractions. Sorting CD133^+^ EVs using magnetic beads provided a specific enrichment of vesicles expressing CD133, that was undetectable in the negative fraction (Fig. 2C). As shown in Fig. 2D and E, the CD133^+^ fraction expressed CD63 at low levels. The proximal tubular markers AQ1 and Megalin, but not Aminopeptidase A were expressed by CD133^+^ EVs. In addition, the glomerular marker CD2AP was highly expressed by CD133^+^ EVs in respect to the negative fraction whereas the collecting duct marker AQ2 was only present in the negative fraction (Fig. 2D and E). These data suggest that CD133^+^ EVs may have a glomerular and proximal tubular origin. CD133^+^ and CD133^−^ EVs did not differ in size, as evaluated by nanoparticle track analysis (Figure S1). Moreover, labelled CD133^−^ and CD133^+^ EVs showed similar binding to latex beads, as proved by similar fluorescence intensity (Figure S1).

We next assessed whether the presence of CD133^+^ EVs within urine might be influenced by parameters such as the urinary flow and urine concentration. After an acute water load, urine (6 h collection) was diluted in respect to a regular water uptake of the same subject (6 h collection); urinary creatinine: 42.3±7.2 g/L vs 119.6±24 mg/dL). The levels of CD133+ EVs did not significantly vary in the same subject according to urine dilution (mean variation 3.8±6.7%, n = 3).

### CD133^+^ urinary Evs are decreased in transplanted patients with SGF

**Figure 3:**
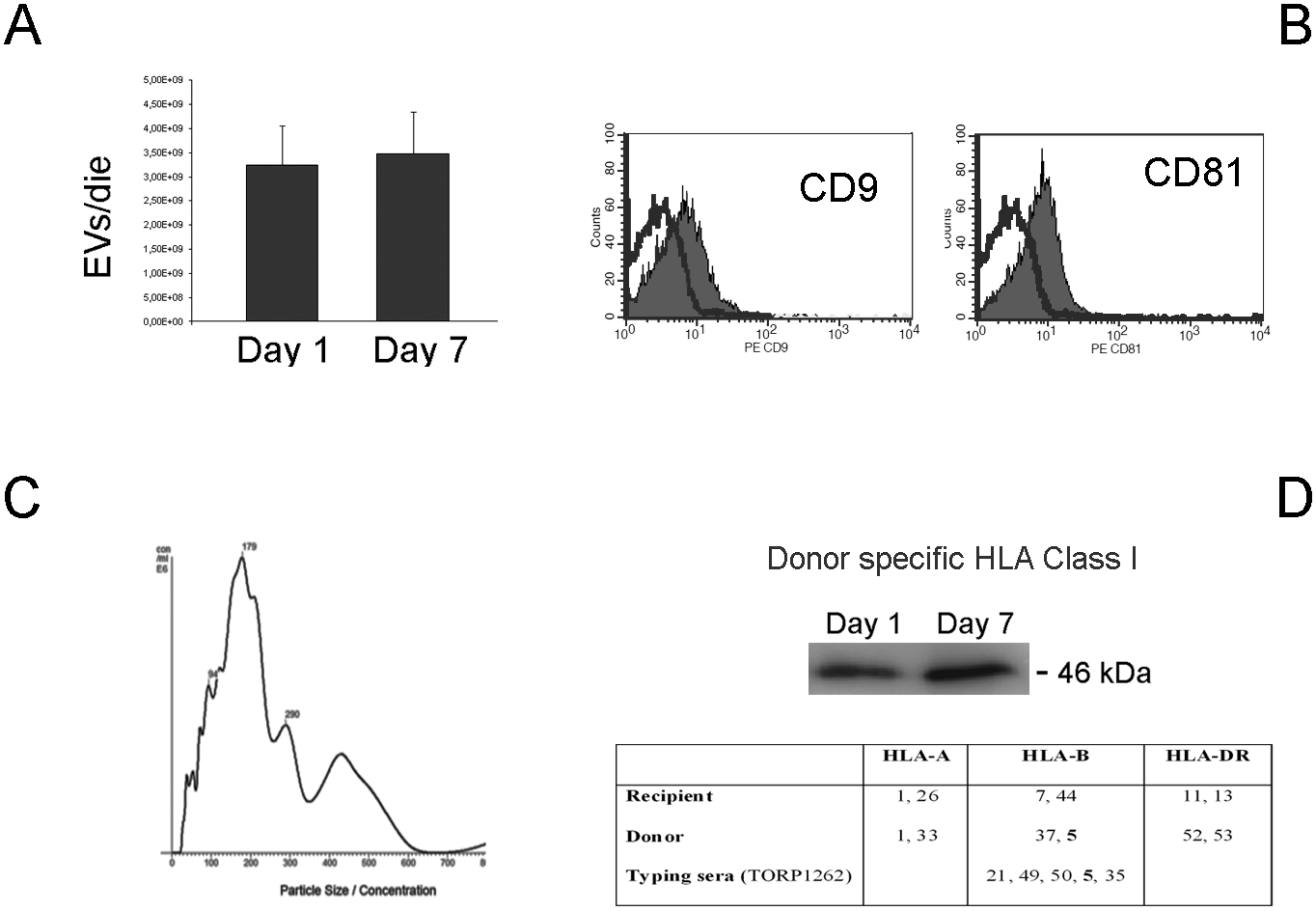
Characterization of urinary EVs in transplanted patients. Urinary EVs were obtained from patients (n = 25) undergoing renal transplant. **Panel A**. Mean number of EVs/day in the urine of transplanted patients were similar at day 1 and 7 after transplant, counted as described in Material and Methods. Data are mean ± SD of all samples. **Panel B and C**. Characterization of EVs showing expression of CD9 and CD81 exosome markers and NanoSight analysis of size distribution profile. Similar results were obtained for samples from all transplanted patients at day 1 and 7. **Panel D**. Representative western blot analysis showing the donor origin of the extracellular vesicle using typing sera containing antibodies directed against HLA class I antigens of the donor not shared by the recipient, at 1 and 7 days after transplant. In the table are reported the HLA typing of the recipient and the donor and the HLA-antigen recognized by the typing serum (TORP1262). HLA-B5 (bold) is present in the donor and not in the recipient. Three patients were tested with similar results.

We next evaluated the presence of CD133^+^ EVs in the urine of transplanted patients at day 1 and 7 after transplant. As soon as 1 day after transplant, EVs were present in the urine and the production evaluated as EV number/die did not significantly vary within the first 7 days (Fig. 3A). EVs from transplanted patients expressed the exosome markers CD9 and CD81 and a mean size of 179 nm (Fig. 3B and C). To check whether EVs derived from the transplanted kidney rather than from the native kidney or from the lower urinary tract, EVs were stained with anti-HLA Class I Abs recognizing the donor specific antigen isoforms. As shown in Fig. 3D, a band detecting donor specific HLA Class I was present both at 1 and 7 days after transplant.

**Figure 4:**
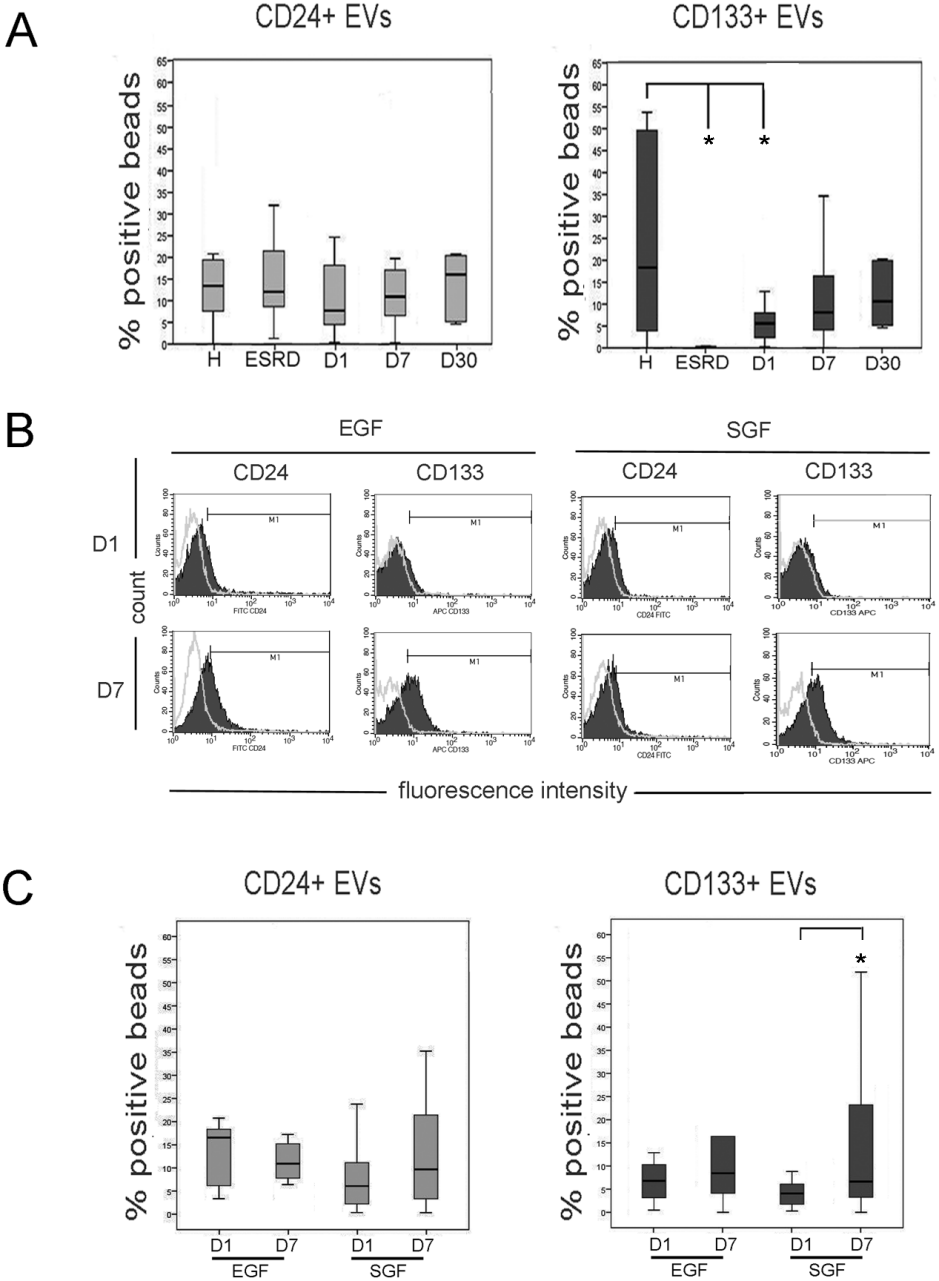
CD24^+^ and CD133^+^ EVs in the urine of normal subjects, ESRD and transplanted patients. Panel A. Levels of urinary CD24^+^ and CD133^+^ EVs in normal subjects (H), ESRD patients with residual diuresis and transplanted patients at day 1, 7 or 30 after transplant (D1, D7 and D30, respectively). A significant reduction of CD133^+^ EVs but not of CD24^+^ EVs was observed in ESRD and transplanted patients at day 1. Data are expressed as % positive beads and are mean ± SD of all samples. **Panel B and C**. Representative cytofluorimetric histograms (B) and median values (C) of CD24^+^ and CD133^+^ EV levels in the urine of patients with EGF (n = 13) and SGF (n = 12) at day 1 and 7 after transplant. Urinary CD133^+^ EVs but not CD24^+^ EVs were lower in EGF patients at day 1, and significantly increased at day 7. Statistical analysis was performed as described in the Materials and methods section. * = p<0.05.

We evaluated the relative percentage of EVs expressing CD133 using a cytofluorimetric analysis (Fig. 4B). The expression of CD24, a marker of urinary vesicles [26], was used as control. First, to assess whether CD133^+^ EVs specifically derived by a functioning kidney, we analyzed CD133^+^ EVs in ESRD patients with a residual diuresis. No CD133^+^ EVs were detected in the urine of ESRD patients. The level of CD24 was maintained constant in healthy subjects and ESRD patients, indicating a specific loss of CD133^+^ EVs in patients with impairment of the renal function.

**Figure 5:**
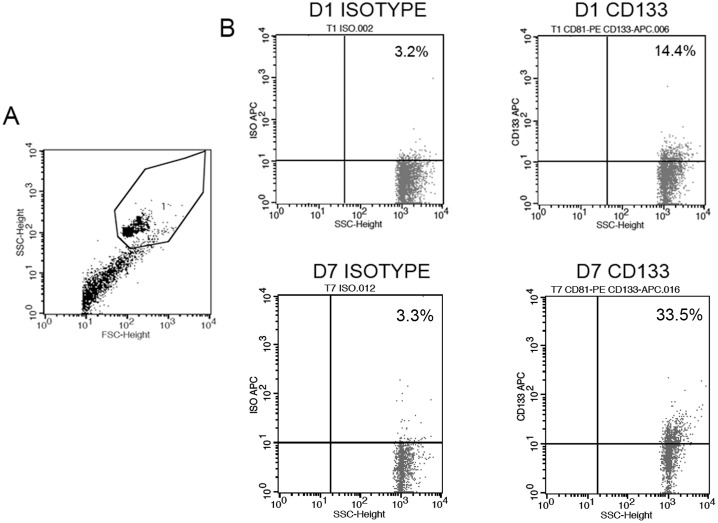
Detection of CD133^+^ EVs in transplanted patients. Panel A and B. Representative dot plots showing the gating strategy of latex bead-conjugated EVs (A) and the fluorescence detection of isotype or CD133 on EVs from a transplanted patients at day 1 or 7.

CD133^+^ EVs were present in patients undergoing transplant in the first day. However, the CD133^+^ EV levels were significantly lower than those of healthy subjects matched for age and sex with the kidney donors. Levels of CD133^+^ EVs increased 7 days after transplant to levels statistically not different from control subjects (Fig. 4A and Fig. 5). In contrast, no significant difference was observed in the levels of CD24^+^ EVs, suggesting the absence of variation in vesicle production by the kidney. Evaluation of CD133^+^ EV levels at 30 days after transplant in 6 patients did not show significant variation in respect to day 7 (Fig. 4A).

Transplanted patients were retrospectively divided in respect to kidney function into patients with EGF or SGF, the latter defined as >3 mg/dl creatinine at day 3 [24]. Interestingly, at day 1 the CD133^+^ EVs were lower in SGF than in EGF patients, although it did not reach a statistical significance (p = 0.08). At day 7, the increase in CD133^+^ EVs levels was significant only in patients with SGF. Levels of CD24, used as control of the presence of urinary vesicles, did not significantly vary in the different conditions (Fig. 4B and C).

The increase of CD133^+^ EVs levels from day 1 to day 7 was not related to different urine dilution, as urine concentration did not significantly vary in the first 7 days post-transplant (urinary creatinine concentration: 34±5 mg/dL at day 1vs 29.8±8 mg/dL at day 7), as recently reported [27]. Moreover, the increase was not dependent on a parallel increase in the glomerular filtration, as the glomerular filtration rate increased from day 1 to day 7 only in EGF but not in SGF patients (Table 1).

### CD133^+^ urinary Evs correlate with the severity of the vascular damage in pre-transplanted kidneys

**Figure 6:**
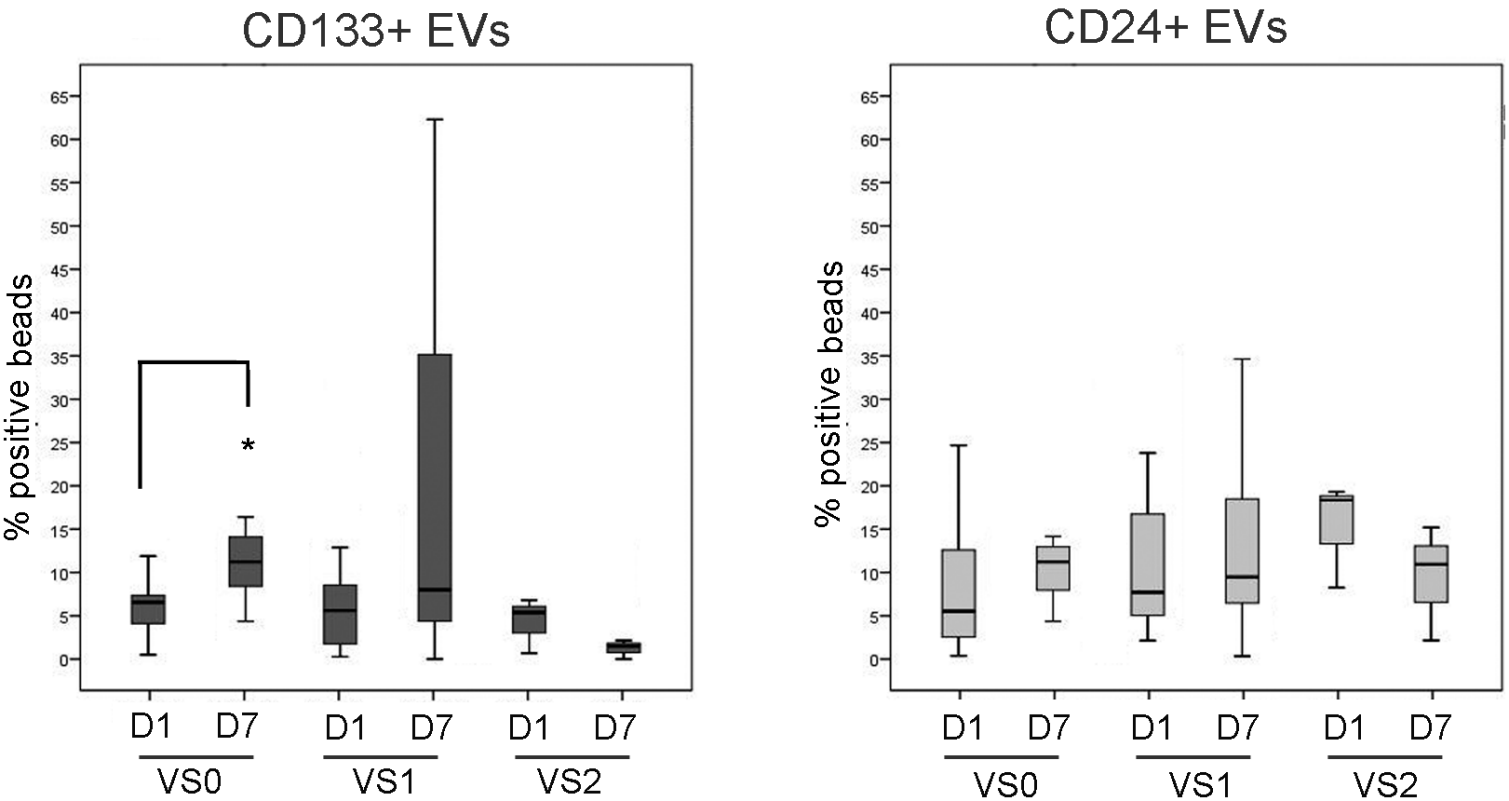
Median values of CD24^+^ and CD133^+^ EV levels in the urine of transplanted patients at day 1 and 7 after transplant (D1 and D7), divided according to the graft vascular lesions (VS), as described in Material and methods. No increase in urinary CD133^+^ EVs was observed in VS2 patients. Statistical analysis was performed as described in the Materials and methods section. * = p<0.05.

In addition, when patients were divided according to the graft vascular lesions, evaluated as indicated in the material and methods [25], CD133^+^ EV levels, and not CD24^+^ EV levels, showed a different increase within time (Fig. 6). In particular, patients with vascular score 2 did not show a recovery of the CD133^+^ EVs levels at day 7 after transplant. No correlation of CD133^+^ EVs was found with other parameters such as ischemia time, introduction of calcineurin inhibitors, HLA mismatch or pre-transplant diuresis (Table 2).

**Table 2:** Correlation of CD133^+^ EVs and patients’ clinical parameters.

		No	CD133^+^ EVs T1	p*	CD133^+^ EVs T7	p*
Ischemia (h)	<15	12	4.38 [0.49–11.88]	0.936	8.41[0.00–56.03]	0.503
	>15	13	6.13 [0.28–31.82]		8.00 [0.00–68.68]	
CNI introduction	ab initio	7	5.37 [0.51–11.88]	0.836	11.23 [1.48–62.29]	0.389
	at Crt<2.5	18	5.86 [0.28–31.82]		7.93 [0.00–68.68]	
HLA Mismatches	<4	12	5.48 [0.28–31.82]	0.852	4.24 [0.00–68.68]	0.087
	>4	13	6.13 [0.49–12.88]		8.69 [1.48–62.29]	
Pre-transplant diuresis	>1 L	11	6.65 [0.28–31.82]	0.085	11.77 [0.34–68.68]	0.166
	<1 L	14	2.81 [0.49–12.88]		6.51 [0.00–62.26]	

No correlation was found among CD133^+^ EVs and ischemia time, introduction of calcineurin inhibitors (CNI), HLA mismatches, and pre-transplant diuresis. Data are expressed are median [min-max], p* = Mann-Withney test.

## Discussion

In the present study, we show that CD133^+^ expressing EVs are present in the urine of normal subjects, but not of patients with ESRD. In the urine of transplanted patients CD133^+^ EVs were present at low levels the first day after transplant, to increase thereafter (at day 7). In particular, at day 1 the level of CD133^+^ EVs was lower in patients showing a slow graft function in respect to those with early graft function, then it significantly increased at day 7. Finally, in patients with a severe pre-transplant vascular damage of the graft, CD133^+^ EVs did not increase at day 7. No significant variation was observed in the presence of CD24^+^ EVs in patients with ESRD or in transplanted patients.

EVs present in urine could derive both from the cell surface or from intracellular multivescicolar bodies and could be accordingly classified as microvesicles or exosomes, respectively. However, a clear difference based on vesicle markers or dimension is at present of debate [28] and, in the present study, we studied the entire population of EVs present in the urine. The presence of CD133^+^ EVs in the urine of normal subject and polycystic kidney disease patients was previously reported using Western Blot and immunogold analysis [20], [29]. CD133^+^/CD63^−^ EVs with a large size (400 nm) were described to be released into body fluids from microvilli of epithelial cells [20]. Other studies reported the biogenesis of CD133^+^ EVs by multivesicular bodies and their release as CD63^+^ exosomes [17], [30]. The results of our study, showing the presence of CD63 at low levels in CD133^+^ sorted EVs by Western Blot analysis, suggests that both type of vesicles may be present in urine. The proteomic analysis of urinary EVs previously identified proteins from renal epithelia extending from the glomerular podocytes through the proximal tubules to collecting ducts [3], suggesting that all nephron segments may contribute to their generation. At variance, the origin of CD133^+^ EVs appeared more restricted to glomeruli and proximal tubules based on the expression of surface markers on CD133^+^ sorted EVs. Further information in support of the renal origin of CD133^+^ EVs is provided by their detection immediately after transplantation. Since patients with end stage renal disease did not release CD133^+^ EVs, the CD133^+^ EVs found in the urine of transplanted patients the first day after transplant are most likely released by the graft. This is also confirmed by the presence of graft-specific HLA Class I antigens by urinary EVs of transplanted patients. The possible function of the CD133^+^ EVs in renal physiology still remains to be investigated. It could be however speculated that CD133^+^ EVs may interact with cells of the nephron, as described for CD133^+^ EVs released in the urine of polycystic kidney disease patients [29].

In the nervous system, the shedding of CD133^+^ EVs from the cell surface was regarded as a mechanism of cell differentiation and of neural specification [16]. Similarly, CD133^+^ EVs are shed by CD133^+^ hematopoietic stem cells during the process of cell differentiation [17]. Our data show that CD133^+^ EVs are continuously released by renal cells suggesting the involvement of CD133^+^ progenitors in the homeostatic turnover of the nephron. The reduction of urinary CD133^+^ EV levels but not of CD24^+^ EVs in end stage renal disease indicates that these vesicles are only released by functioning renal tissue, suggesting an exhaustion of the progenitor activity that parallels the loss of kidney function.

It could be therefore speculated that the number of CD133^+^ EVs may reflect the activity of CD133^+^ cells in the kidney, as CD133^+^ EVs were lower in patients with a slow graft function in respect to those with early graft function. In addition, in these latter patients, CD133^+^ EVs significantly increased after 7 days, possibly underlying that their presence correlates with the graft recovery after tissue damage. Moreover in the same patients the number of CD133^+^ EVs at day 7 reached levels not significantly different in comparison with normal subjects. Our results parallel the data reported by Loverre et al. [13] showing that CD133^+^ cells were reduced in renal tissue of pre-transplant delayed graft function patients in respect to early graft function patients. In addition, in delayed graft function patients, an increase of proliferating CD133^+^ cells was observed 10 days after transplant [13]. These data may further implicate CD133^+^ cells in the regeneration of renal tissue after injury due to cold ischemia and ischemia-reperfusion, as suggested in several studies [14], [15], [31]. In addition, in patients with a severe pre-transplant vascular damage, we observed the lack of increase in urinary CD133^+^ EVs at day 7. This may imply an inefficient regeneration in the renal tissue of patients with high vascular damage. The lack of variations in CD24^+^ EVs, used as marker of urinary EVs, indicates that the observed differences in CD133^+^ EV release do not reflect alteration in EV number but rather in the composition of the vesicle population. Indeed, the relative CD133^+^ EV levels did not vary in relation to urine concentration or glomerular filtration rate.

Previous studies identified that the amount of membrane particle-associated CD133 was significantly enhanced in patients with glioblastoma and in epileptic patients as compared to healthy adults [18], [19]. At variance with these studies, evaluating the amount of CD133 using Western Blot analysis, our study was based on cytofluorimetric evaluation of CD133^+^ EVs labeled on latex beads. This is a common method to analyze the expression of markers expressed on the EV surface [23]. This method resulted reliable and highly sensitive. In addition, as the results represent a relative expression of positive EVs on the total population, it was not influenced by urine dilution and EV amount in the urine. We are, however, aware that our data do not represent real percentages of CD133^+^ EVs, as the level of positivity is the result of numerous EVs bound to each bead. These data support the idea that the analysis of urinary CD133^+^ EVs may represent a possible biomarker for tissue injury or for the repair capacity of the nephron.

The possible use of urinary exosomes as markers of renal damage has already been proposed. For instance, activating transcription factor 3 and Wilms tumor 1 were associated with early tubular and podocyte injury in different experimental models [32]. Moreover, a reduction in the levels of AQ1 in urinary exosomes has been associated with renal ischemia–reperfusion injury in rats [33]. Finally, it has been recently shown that detection of fetuin-A in urinary EVs is a biomarker of renal injury in patients developing acute kidney injury [34]. The evaluation of urinary CD133^+^ EVs as marker of tubular function and repair may be of interest in acute renal injury, and it could be possibly extended to patients with different levels of chronic renal damage.

Taken together, these results suggest that CD133^+^ EVs may reflect the activity of CD133^+^ progenitor cells in renal homeostasis and may provide information on their regenerative potential after acute kidney injury.

## Supporting Information

Figure S1 (PDF)
