# Supplementary material for: Urinary CD133+ Extracellular Vesicles Are Decreased in Kidney Transplanted Patients with Slow Graft Function and Vascular Damage
Source: PLoS One. 2014 Aug 6;9(8):e104490. doi: 10.1371/journal.pone.0104490 (PMC4123993; doi:10.1371/journal.pone.0104490)
Supplement: Figure S1 — (PDF) [file pone.0104490.s001.pdf]

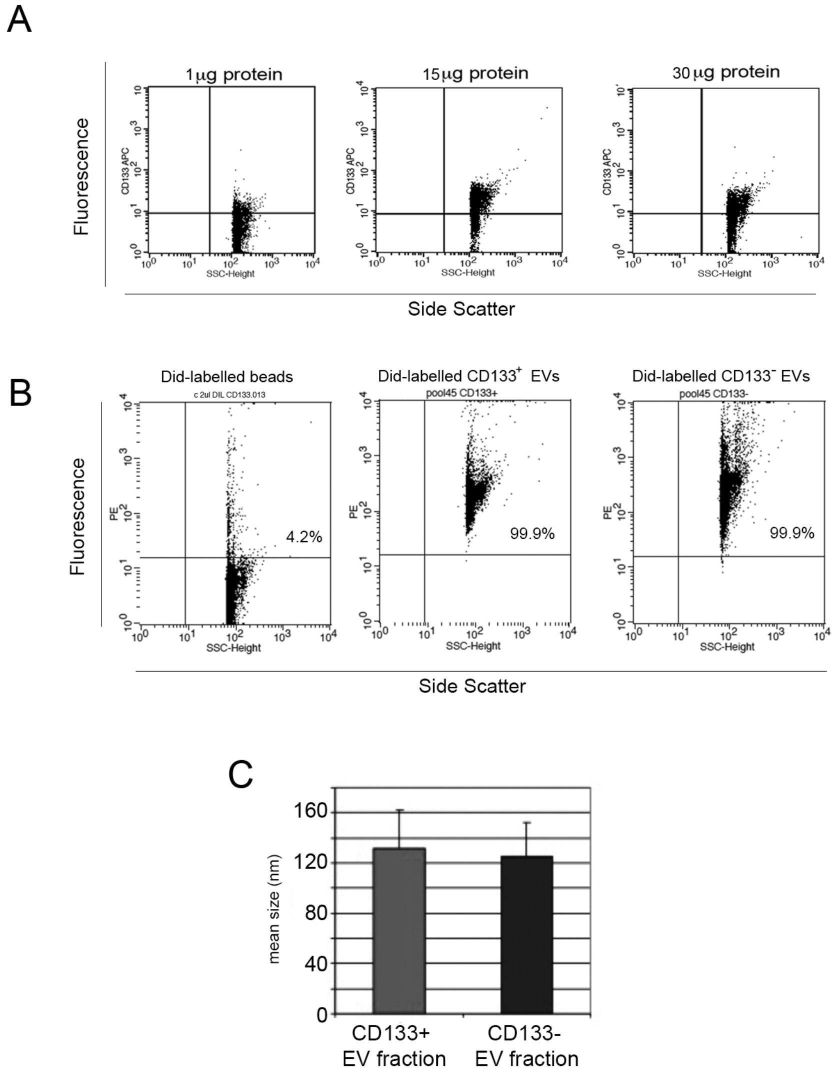

Supplementary Figure 1

Figure S1. **Cytofluorimetric controls of EV dosage and binding onto latex beads**. **Panel A**. Representative dot plots showing fluorescence detection of CD133 at increasing concentrations of urinary EVs. No increase in signal was obtained after 15 µg protein. **Panel B.** Representative dot plots showing fluorescence detection of EVs labelled with Did-fluorescent dye. No difference in fluorescence was observed between the $CD133^+$ and the $CD133^-$ sorted fractions. The first plot

shows labelling of latex beads alone, as control. **Panel C**. NanoSight analysis of EV mean size showing no difference in CD133$^+$ and the CD133$^-$ sorted fractions.
